# High-Risk Advanced Maternal Age and High Parity Pregnancy: Tackling a Neglected Need Through Formative Research and Action

**DOI:** 10.9745/GHSP-D-17-00417

**Published:** 2018-06-27

**Authors:** Khadidiatou Ndiaye, Erin Portillo, Dieneba Ouedraogo, Allison Mobley, Stella Babalola

**Affiliations:** a George Washington University, Washington, DC, USA.; b Johns Hopkins Center for Communication Programs, Baltimore, MD, USA.; c Centre International de Formation en Recherche Action, Ouagadougou, Burkina Faso.; d Independent consultant, Baltimore, MD, USA.

## Abstract

Pregnancy among women of advanced maternal age (those 35 years or older) or among women of high parity (those having had 5 or more births) is linked to maternal and infant mortality. Yet little is known about the drivers of these pregnancies as they are often neglected in existing family planning and reproductive health programs. To better understand the context in which advanced maternal age and high parity pregnancies occur and the acceptability of discussing associated pregnancy risks, the Health Communication Capacity Collaborative (HC3) conducted formative qualitative research between January and March 2015 on the perception and determinants of such pregnancies in rural and urban areas of Niger and Togo. We supplemented this research with secondary analyses of data from Demographic and Health Surveys and a 2014 Niger survey. Our formative research showed that urban Togolese participants demonstrated more knowledge about advanced maternal age and high parity pregnancy risks than did participants in Niger as a whole. We found that such pregnancies were generally seen as part of reproductive norms in situations where fertility rates remain exceptionally high, especially in Niger. Social, gender, and religious norms, including competition between co-wives, also drove women into high parity and advanced maternal age situations, particularly in Niger, highlighting the need to bring men and community and religious leaders into family planning conversations to increase acceptance. The study also provided important insights needed to address these high-risk pregnancies through culturally appropriate health communication interventions. A main insight was that providers often had incomplete information about advanced maternal age and high parity pregnancies and lacked communication skills, protocols, and tools to appropriately discuss such pregnancy risks with clients. HC3 used these and additional findings to create an Implementation Kit (I-Kit) for family planning and maternal and child health program managers with guidance and tangible tools to address advanced maternal age and high parity pregnancy through social and behavior change communication. The I-Kit includes health communication materials to engage women, men, decision makers, communities, health care providers, journalists, and others. In 2016 and 2017, one organization each in Niger and Togo piloted the I-Kit, integrated selected I-Kit tools into their unique programs, and documented their experiences. Both organizations credited the I-Kit with expanding the scope of their programs to now address advanced maternal age and high parity pregnancy and provided concrete suggestions for adapting the materials according to activity and intended audience.

## Abstract (precis)

Harmful social norms and lack of knowledge contribute to risky pregnancies in older and high-parity women in low- and middle-income countries. A social and behavior change communication resource combining technical guidance with tangible client and provider materials was designed to address and prevent such pregnancies in Niger and Togo.

Résumé en français à la fin de l'article.

## INTRODUCTION

Family planning remains a key aspect of the global health agenda. Following a decline in global funding in the late 2000s, family planning has regained momentum and international attention in recent years.^1^ This funding resurgence has been coupled with renewed governmental commitments and global advocacy. For example, building upon the 2012 London Summit on Family Planning, the Family Planning 2020 (FP2020) movement was established to champion global advocacy and drive country-level support for family planning. Furthermore, while Goal 3 of the Sustainable Development Goals (SDGs) includes a specific target to “ensure universal access to sexual and reproductive health services, including for family planning,”^2^ the argument has also been made that investing in family planning will accelerate achievement across all 5 SDG themes.^3^

While these efforts have increased family planning programs in developing countries, there remain distinct neglected needs, risk factors, and population segments. For example, while much attention is given to preventing pregnancy among women before age 18, increasing voluntary contraceptive uptake, and establishing healthy spacing intervals between pregnancies,^4^^–^^6^ less focus is typically placed on addressing pregnancies among women of advanced maternal age (those 35 years or older) or high parity (having had 5 or more births)—even though these pregnancies are high risk and linked to maternal and infant mortality.

Family planning programs typically place less attention on addressing pregnancies among women of advanced maternal age or high parity.

Advanced maternal age and high parity pregnancies are prevalent in sub-Saharan African countries where parity rates are high and childbearing often continues until menopause.^7^ A 29-country study found that advanced maternal age pregnancies “significantly increased the risk of maternal adverse outcomes, including MNM [maternal near miss], MD [maternal death], and SMO [severe maternal outcome], as well as the risk of stillbirths and perinatal mortalities.”^8^ “Maternal near miss” refers to cases “in which women present potentially fatal complications during pregnancy, delivery or during the puerperium, and who survive merely by chance or by good hospital care.”^9^ High parity complications include anemia in the mother, postpartum hemorrhage, and fetal malpresentation. It is important to also consider that a high parity pregnant mother may also be of advanced maternal age, and hence her risks are compounded—and may be made graver still if her pregnancies are spaced too closely.

A review of the literature shows that while advanced maternal age- and high parity-specific research exists, much is from high-income settings in the West. Only a few studies from sub-Saharan Africa exist,^10^^,^^11^ and most of this work focuses on establishing risks with little to no research on knowledge, attitudes, and behaviors relating to such pregnancy.^12^ Understanding how a country's culture and context influence individuals' and communities' beliefs and practices relating to these pregnancies is also crucial in developing effective interventions to address them.

Given the risks and region-specific information gaps, the Health Communication Capacity Collaborative (HC3) project of the Johns Hopkins Center for Communication Programs conducted formative research and created and piloted a Healthy Timing and Spacing of Pregnancies: Addressing Advanced Maternal Age and High Parity in Family Planning Programs Implementation Kit (I-Kit) (https://sbccimplementationkits.org/htsp/) to help program managers address these high-risk pregnancies in their programs using social and behavior change communication.

## RESEARCH GOALS AND OBJECTIVES

To understand what drives these high-risk pregnancies, we conducted formative research in 2 countries with considerable advanced maternal age and high parity rates: Niger and Togo. The countries represent 2 different contexts. Niger, with a largely Muslim population, generally has more conservative social and religious norms and a larger rural population than Togo, and women in Niger desire more children than they have. This is the opposite of the situation in Togo (Table 1).

**TABLE 1.** Key Indicators for Niger and Togo

	Niger	Togo
Urban population (% of population living in urban areas)	18%	40%
Polygamy (% of married women in polygamous marriage)	36%	32%
Total fertility rate	7.6	4.8
Urban	5.6	3.7
Rural	8.1	5.7
Ideal number of children (among women)	9.2	4.3
Urban	7.4	3.6
Rural	9.6	4.9
Advanced maternal age (% of all women 35–49 who had a child at 35 years or older)	60%	46%
High parity (% of all women who had 5 or more births)	43%	22%

Source: Enquête Démographique et de Santé et à Indicateurs Multiples du Niger 2012^13^; Enquête Démographique et de Santé au Togo 2013–2014.^15^

We focused on Niger and Togo for several reasons. First, for more than a decade, Niger has had the highest total fertility rate in the world, currently at 7.6—an increase from 7.0 and 7.2 in past Demographic and Health Survey (DHS) reports.^13^ On average, women in urban Niger think having 7.4 children is ideal while their rural counterparts aspire to have 9.6 children. The mean ideal number of children for women in Niger has increased over the years, from 8.2 in 1992 to 9.2 in 2012.^14^ Although Togo's fertility rate is lower than Niger's and is showing a steady decrease from 6.4 over the past 25 years, Togo's most recent total fertility rate of 4.8 still demonstrates high parity risk for women in the country, especially in rural areas.^14^ Women in Togo have on average 3.7 children in urban areas and 5.7 in rural locations, and in both urban and rural areas women say their ideal number of children is fewer than the actual number of children they have, at 3.6 and 4.9, respectively.^15^ The mean ideal number of children for women in Togo has decreased over the years, from 5.3 in 1988 to 4.3 in 2013/2014.^14^ Per each country's most recent DHS, 43% and 22% of women had 5 or more births in Niger and Togo, respectively (Table 1). These same reports show the ideal number of children is higher among men than women in each country: currently married men in Niger desire 12.4 children, and in Togo, 5.4 children. This, too, has been increasing in Niger and decreasing in Togo over the past 25 years. High fertility rates and high parity status are also coupled with early childbearing in both countries, which carries with it its own significant maternal and infant morbidity and mortality risks. Early childbearing is more common in Niger, where 40.4% of women ages 15 to 19 are mothers or pregnant with their first child. This statistic is much lower in Togo, where 16.5% of women between ages 15 and 19 have begun childbearing.^14^

Another reason we focused on Niger and Togo is that both countries have critical family planning needs. Contraceptive use is relatively low in both Niger (14% of married women)^13^ and Togo (20% of married women).^15^ In Niger, the percentage of women with an unmet need for modern contraceptive methods has increased over the past 5 years from 18.7% to 20.8%; 41.9% of women's demand is satisfied with a modern contraceptive method.^16^ In Togo, 34.6% of women have an unmet need for a modern method of family planning—a rate that has decreased over the past 5 years, and 40.9% of women's family planning demand is satisfied with a modern method.^17^ Advanced maternal age is particularly common in both countries. According to each country's most recent DHS, 60% of women in Niger and 46% of women in Togo had a child at age 35 or older.^13^^,^^15^

Finally, both countries have NGO and government support for family planning programs. In addition to being FP2020 focus countries, both Niger and Togo are members of the Ouagadougou Partnership. The Government of Niger has committed to increase the contraceptive prevalence rate to 50% in 2020, from 10.8% in 2012. The Niger government plans to achieve this by increasing contraceptive availability (e.g., through mobile and community-based distribution), strengthening demand for family planning methods and services (e.g., through social marketing and information and education communication efforts), and creating a more favorable family planning environment through policy and other structural efforts.^18^ The Government of Togo has committed to increase contraceptive prevalence to 35.5% in 2022, primarily by creating demand for family planning, increasing availability of and access to services, strengthening contraceptive commodity procurement to avoid stock-outs, and creating an enabling finance and policy environment.^19^ The contraceptive prevalence rate in Togo has increased from 13.2% in 2012 to 23.2% in 2017, which was the highest prevalence among all 9 Ouagadougou Partnership countries.^20^

The overall goals of the formative research were to: (1) understand the knowledge, attitudes, and behaviors that contribute to advanced maternal age and high parity pregnancy incidence/prevalence, and (2) understand how the findings could be used to improve maternal and child health and family planning programs through a pilot intervention focused on social and behavior change communication.

## METHODS

The formative, qualitative research took place between January and March 2015 in 1 urban and 2 rural locations in Niger and Togo (Table 2). The selection of study sites was based on several factors including fertility rate, prevalence of advanced maternal age pregnancies, cultural diversity, and level of contraceptive use as well as accessibility from the capital. Selecting sites closer to capital cities is one limitation of the study, as knowledge, attitudes, and behaviors prevalent among populations living near a capital city may not be representative of those of more remote communities. Further, participants in each site were presumed to live in each location, rather than having traveled to research sites to participate in the study. As such, findings should be interpreted as specific to populations living in each site and cannot be assumed to be representative of prevailing knowledge, attitudes, and behaviors throughout each country.

**Figure:**
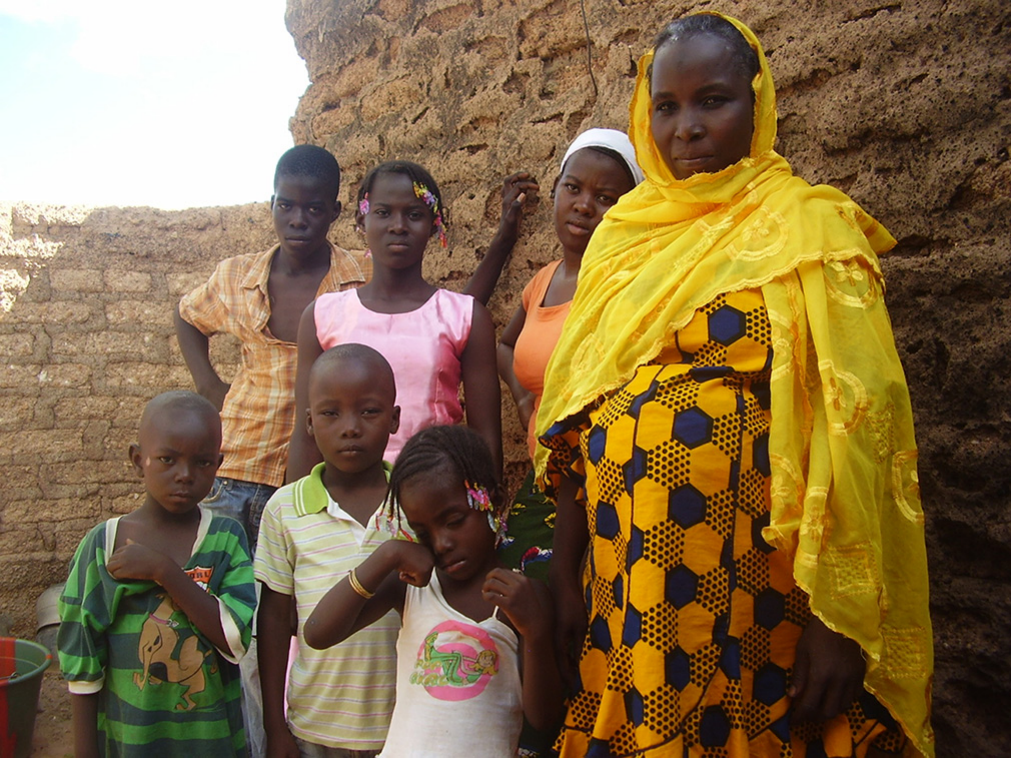
A woman in West Africa with her 6 children. High parity pregnancies can lead to complications including anemia in the mother, postpartum hemorrhage, and fetal malpresentation. © 2014 Dieneba Ouedraogo.

**TABLE 2.** Number of Participants (and Groups) by Qualitative Research Method and Location

	Niger			Togo			Total
	Niamey (urban)	Koygoro (rural)	Mokko (rural)	Lomé (urban)	Aouda (rural)	Adjengre (rural)	
Focus group discussions							
Women	36 (4)	8 (1)	8 (1)	31 (4)	19 (2)	25 (2)	127 (14)
Male partners	24 (3)	8 (1)	8 (1)	25 (3)	8 (1)	9 (1)	82 (10)
Mixed (men and women)	8 (1)	–	–	8 (1)	–	–	16 (2)
Case studies	2	1	1	2	1	1	8
In-depth interviews							
Service providers	3	2	1	3	2	2	13
Couples	8 (4)	4 (2)	4 (2)	4 (2)	4 (2)	4 (2)	28 (14)
Community leaders	2	2	1	2	2	2	11
Total							285

In Niger, we conducted the study in Niamey (urban), Koygoro (rural), and Mokko (rural) in the Dosso region, which is approximately 130 km from the capital city of Niamey. Villages in the north of the Dosso region were selected due to safety concerns. In Togo, we conducted the study in Lomé (urban), Aouda (rural), and Adjengre (rural) in the Plateaux region, located 169 km from the capital Lomé. This region was chosen because of its mix of religions (Muslim, Christian, and indigenous religions). We conducted focus group discussions, case studies, and in-depth interviews with 285 (174 female, 111 male) health care service providers, women of advanced maternal age and/or high parity, male partners of women in these risk categories, and community leaders. In each study site:

- **Focus group discussions** were conducted with women of advanced maternal age and/or high parity and with male partners of women in these risk categories to gather data about collective perceptions and attitudes that influence choices about reproduction, particularly advanced maternal age and/or high parity pregnancies.
- **Case studies** were collected from individual women of advanced maternal age and/or high parity who had difficult pregnancies or deliveries. These histories highlighted knowledge and attitudes of these women about risks with such pregnancies and how these impacted their pregnancies and deliveries.
- **In-depth interviews** were conducted with:
  - **Advanced maternal age and high parity couples** to understand how marriage, gender dynamics, and individual, cultural, economic, and other factors impacted fertility desires and reproductive health decision making regarding advanced maternal age and high parity pregnancies.
  - **Maternal and infant health service providers** to understand their perception and knowledge of advanced maternal age and high parity pregnancies, and how they communicated with their clients about these types of pregnancies.
  - **Community leaders** to understand their maternal health and family planning perspectives, as well as their view of advanced maternal age and/or high parity pregnancies.

We supplemented the qualitative research with data from the “Customer Insights Research for Family Planning Demand Generation in Niger,” a nationwide survey of 2,000 women between the ages of 15 and 49, conducted in 2014 by Hope Consulting (which merged with SwitchPoint to form Camber Collective in July 2015).^21^ We analyzed a subset of this survey data, the responses of the advanced maternal age and high parity women (n=760), to examine specific knowledge, attitudes, and behaviors related to advanced maternal age and high parity pregnancies. In this article, we refer to this subset analysis as the Niger survey. Additionally, we performed secondary analyses on select DHS indicators from Niger^13^ and Togo.^15^

The research protocol was approved by the Johns Hopkins University Institutional Review Board (IRB) and by local IRBs in Niger and Togo. We conducted focus group discussions and in-depth interviews in Zharma and Hausa languages in Niger and in Mina and Kabiye languages in Togo; all were recorded and transcribed in the field into French. A resource person (often the main facilitator who spoke the language in which the interview was conducted) checked and evaluated the transcripts. Finally, we conducted content analysis using Microsoft Word and coded responses thematically.

## FINDINGS

### Perceived Prevalence of Advanced Maternal Age and High Parity Pregnancies

Participants in both rural and urban Niger believed advanced maternal age pregnancies were common in their communities. In Togo, responses were mixed; while most viewed such pregnancies as a “rural problem.” others reported they were also prevalent in cities.

In Niger, most participants reported high parity was common in both urban and rural settings. One man from urban Niger explained in an in-depth interview:

*Personally, 5 children is good. But if God arranged for us to have more, that would not be a problem.*

The Niger survey confirmed the participants' perceptions that high parity was common, showing 42% of women between the ages of 15 and 49 in a relationship at the time of the survey were high parity. Among these, 71% said they wanted more children. In Togo, participants generally acknowledged the high number of high parity pregnancies in their country, but were divided about high parity pregnancy frequency in urban areas.

### Risk Perceptions

In Niger, participants saw pregnancy itself as a perilous situation for women but did not perceive age or parity to compound the risk. Those who did associate dangers with advanced maternal age and high parity pregnancies did so generally, and referred mostly to the death of the mother and, secondarily, to that of the baby. These 2 risks were perceived as the most common and the most serious. Togo participants were somewhat more aware of age- and parity-related risks and were also concerned about advanced maternal age and high parity women dying as a result of pregnancies. They mentioned infant mortality, the likelihood of genetic defects, and even social consequences of such pregnancies.

Participants in Niger recognized pregnancy as a perilous situation for women but did not perceive age or parity to compound the risk.

### Religious Beliefs and Cultural Norms

We found that religious beliefs contributed to advanced maternal age and high parity pregnancy, particularly among Muslim participants in both countries, and more prominently in Niger than in Togo. Many believed Islam forbids any interference with reproduction and mandates that women have the number of children “God gave them,” regardless of their desired fertility. Religion was scarcely mentioned as a factor among non-Muslim participants.

In both countries, male and female participants reported an unfavorable cultural norm toward limiting births or did not feel it was their place to prevent births. These norms were stronger in Niger and in rural Togo. One man from urban Niger, who did not use family planning, explained:

*Really it's not good to limit births to 3, 4, or 5 children. It's not our culture. So those of us who have 4 wives—and if we only want 4 children? So every woman will stop after a single child? (Hum!) In any case, we would like every woman to have 16 children. … Really, [limiting births] is not normal, [and] not just in Niger.*

### Perceived Benefits of Large Families

In Niger, and to a lesser extent in Togo, participants felt having a large family helped them to be perceived positively and as “blessed by God” in their community. Participants also felt that having more children added to a family's monetary wealth, ensuring that parents would be cared for in their old age. Finally, participants in both countries favored large families because of perceived high infant mortality rates; the thought is to give birth to many children in the hopes of always having some children should others succumb to illness or death. According to each country's DHS, however, infant mortality rates have *decreased* sizably from 123 per 1,000 live births in 1998 to 51 in 2012 in Niger, and from 80 in 1998 to 49 in 2013/2014 in Togo.^14^ In comparison, fertility rates in Niger *increased* from 7.2 to 7.6 between 1998 and 2012, and have dropped only slightly in Togo from 5.2 to 4.8 between 1998 and 2013/2014. In urban Togo, we did see evidence that norms are shifting toward acceptance of and desire for using family planning to have smaller families.

Participants in Niger and Togo favored large families because of perceived infant mortality rates, yet infant mortality has decreased sizably in both countries.

### Role of Polygamy, Early Marriage, and Marital Instability

More common in Niger than in Togo, our research revealed women in polygamous situations feared real consequences if they had too few children—fears that some men confirmed. One man from urban Togo explained in a focus group discussion:

*If you do not want to raise your hands to implore God because your husband wants to take another wife, you must agree to lift your legs. Yes, if the woman wants to close her legs instead of providing all the children she can have, the man will want to take a second wife. If she doesn't want him to take a second wife, she is forced to open her legs. That's why instead of raised hands “alolédji” it's instead lifted legs “afolédji,” you see?*

Having many children therefore served to (1) prevent the husband from attempting to take another wife or (2) have a competitive edge over co-wives for the husband's attention, resources, and eventual social status and inheritance should the husband die.

Niger has the highest rate of child marriage in the world, with 76% of girls marrying by age 18,^22^ and DHS lists the median age at first marriage at 16. In Togo, 22% of girls are married by age 18,^23^ and the median age at first marriage per the DHS is 20. In both countries, our research showed early entry into a relationship increased the number of children a woman had when limiting births was not allowed. Once married, women lacked acceptable grounds to delay childbearing. In addition to early marriage, participants reported that divorce and remarriage also put women in circumstances where, regardless of age or parity, they had to provide children to their new spouse.

### Health Care Provider Practices

Interviews with maternal and infant health care professionals in both countries revealed inconsistent and unstructured communication with clients about advanced maternal age and high parity pregnancy risks. Providers had low or very general knowledge about age- and parity-related complications, though knowledge levels were acceptable among midwives compared with community health workers and other lower-level cadre providers, who demonstrated a poor understanding of advanced maternal age and high parity pregnancy risks. Further, providers reported that no guidelines existed on when or how to discuss advanced maternal age and high parity pregnancy with clients, and lamented a lack of materials to support such counseling. Finally, providers seemed to lack the skills needed to communicate risk in culturally appropriate ways. This sometimes led clients to fear or mistrust providers. One service provider in rural Togo told of a particular health center where women no longer wanted to visit. Women believed that when a particular midwife at the facility discussed potential pregnancy complications with women, she was wishing misfortune on them, which would then surely come true.

Providers in Niger and Togo had low or very general knowledge about age- and parity-related pregnancy complications.

## PROGRAMMATIC IMPLICATIONS

Overall, the study showed that urban locations had more accepting cultural norms about family planning use compared with rural locations and that urban participants in Togo demonstrated more knowledge about advanced maternal age and high parity pregnancy risks than Niger participants as a whole. We found such pregnancies are generally seen as part of reproductive norms, and limiting is forbidden in contexts where fertility rates remain exceptionally high, such as in Niger. In some urban settings, however, particularly in Togo, these norms were shifting as some “positive deviant” men and women were recognizing the social, health, and economic value of planning pregnancies and having smaller families. Pregnancy risks, such as the death of the mother or the child, were key fears among men and women in both countries. In Togo, these were already understood by some to be elevated risks in advanced maternal age and high parity pregnancy. However, these risks were inconsistently or poorly communicated at the service delivery level.

Health communication is an indispensable tool for increasing understanding about advanced maternal age and high parity pregnancy prevalence and risks, and it is key to catalyzing improved behaviors and strengthening existing family planning programs. The study findings suggest clear opportunities to:

- Advocate for addressing advanced maternal age and high parity pregnancy risks in national health agendas and developing data-driven, comprehensive communication strategies accordingly. Such strategies and associated messaging should be designed to address a country's specific context—for example, according to attitudes about spacing and limiting pregnancies, prevailing marriage dynamics (such as polygamy), and prevalence of advanced maternal age compared with high parity.
- Increase capacity among providers who interact with clients on maternal and child health and family planning matters, including community health workers, midwives, and facility-based providers, to communicate advanced maternal age and high parity pregnancy risks by improving counseling and clinical skills during pre- and in-service trainings.
- Include advanced maternal age and high parity pregnancy information in maternal, newborn, and child health programs, including child health and immunization visits, and in family planning programs, including postpartum family planning programs. This way, programs can better reach clients at a time when they may be thinking about current family size and a future pregnancy, or family health, respectively.
- Work with local organizations and structures, including religious leaders, to develop community-centered programs that address social, religious, and cultural norms that perpetuate advanced maternal age and high parity pregnancy (e.g., polygamy and early marriage), and emphasize positive, existing norms (e.g., prioritizing the health of the mother and spacing births) through proven communication strategies.
- Engage male partners (e.g., through counseling and community activities) to understand advanced maternal age and high parity pregnancy risks and help prevent such pregnancies in their households, where strict gender roles elevate men as family planning decision makers.
- Develop health communication tools specific to advanced maternal age and high parity pregnancy for women's health gatekeepers to promote awareness, change attitudes (e.g., regarding risk factors, modern contraceptive methods, and the decline in infant mortality), and catalyze lifesaving behavior change among key audiences.

## TURNING RESEARCH INTO PRACTICE

With many of these implications in mind, we created the Healthy Timing and Spacing of Pregnancies I-Kit, focused exclusively on addressing advanced maternal age and high parity pregnancy risks. The I-Kit is adaptable, with source files (in Microsoft Word, InDesign, etc.) available upon request. The I-Kit is designed to save program managers the time and money of creating materials on advanced maternal age and high parity from scratch and to enable them to expand their projects' breadth and impact by including communication activities on such pregnancies in their existing family planning and maternal and child health work. The I-Kit is grounded in the formative research findings described in this article, and includes a guide for program managers that explains how to integrate risk information, key messages, and calls to action into existing relevant projects using social and behavior change communication theories and processes. It also includes a collection of ready-to-use or adaptable health communication tools including client brochures, a community mobilization guide, counseling assessment guides, a provider poster, a guide for researchers, a guide for journalists, and infographics.

We developed the Healthy Timing and Spacing of Pregnancies Implementation Kit to address advanced maternal age and high parity pregnancy risks.

In 2015, the I-Kit was pretested in Niger and Togo with advanced maternal age and high parity women, male partners, community and facility health workers, journalists, program implementers, and other NGO and government representatives to gauge the materials' clarity, cultural appropriateness, and usefulness. We conducted focus groups, working sessions, user observations, and interviews. Based on participant feedback, we revised resources to trim text, add or adjust images for more conservative audiences, and adjusted translations to use preferred and more common or regionally acceptable terminology. We also expanded certain resources to be more inclusive of men, youth, religious leaders, community health workers, and TV and print (rather than just radio) journalists. The I-Kit was finalized in 2016. We then contracted with Marie Stopes International (MSI) in Niger and the Association Togolaise pour le Bien-Être Familiale (ATBEF) in Togo to pilot selected I-Kit elements and tools and identify opportunities to adapt the I-Kit's materials based on on-the-ground use.

The Implementation Kit was pretested in Niger and Togo with women, male partners, community and facility health workers, journalists, program implementers, and other NGO and government representatives.

MSI, active in Niger since 2013, provides quality reproductive health services in and around Maradi, Niamey, and Tillaberi. MSI operates primarily through its Niamey clinic and a series of mobile outreach workers, including mobile clinic teams, social mobilization agents, and community-based promoters. ATBEF is a member of the International Planned Parenthood Federation and has been delivering sexual and reproductive health services throughout Togo since 1975. ATBEF operates through 5 clinics, 2 mobile teams, and a cadre of community health workers.

To prepare for the pilot, each organization reviewed the entire I-Kit and chose specific tools to incorporate into existing project activities to share advanced maternal age and high parity information with their clients and communities. Table 3 outlines each organization's selections and their implementation periods, which ranged from 4 to 7 months based on each organization's staff availability and activity schedules. We provided modest financial support and delivered technical assistance via phone, Skype, and email. The technical assistance culminated with 1 in-person country visit each. Time and financial resources allowed each organization to print, organize trainings, and disseminate the I-Kit materials as is, and we asked that MSI-Niger and ATBEF follow each material's use closely, documenting successes and challenges along the way.

**TABLE 3.** Healthy Timing and Spacing of Pregnancies I-Kit Elements Implemented by Pilot Partner Organizations and Time Period of Each Pilot

MSI-Niger July to October 2016	ATBEF September 2016 to March 2017
Implementation manual for program managers	Implementation manual for program managers
Client brochure for more conservative audiences	Client brochure for less conservative audiences
Counseling and assessment guide for providers	Counseling and assessment guide for providers
Counseling and assessment guide for community health workers	Counseling and assessment guide for community health workers
Reminder poster for facility-based providers	Reminder poster for facility-based providers
Journalist guide	Infographics for policy and decision makers

Abbreviations: ATBEF, Association Togolaise pour le Bien-Être Familiale; I-Kit, Implementation Kit; MSI, Marie Stopes International.

Both MSI and ATBEF initiated the pilot by holding a staff and stakeholder workshop to review the program manager guide, and orient their teams on advanced maternal age and high parity pregnancy, why it should be a priority, and how to address it through social and behavior change communication. These types of communication activities were somewhat new to both organizations, arguably more so to ATBEF. Despite this initial challenge—surmounted through virtual technical support sessions and additional French-language social and behavior change communication references—each organization successfully employed the I-Kit tools into their work.

Overall, MSI reached 12,757 women and men with the pilot activities through mobile clinic outreach, community discussions, individual counseling, and workshops with journalists. ATBEF reached 3,337 individuals through client counseling and community education sessions and provider and community health worker training sessions. These numbers are impressive both considering the short implementation period, but particularly when considering that for most, if not all, beneficiaries, this was the first time receiving complete and correct information about advanced maternal age and high parity pregnancy risks (Supplements 1 and 2).

Specific achievements in Niger included 2 news stories, one each on local television and print outlets, spurred by a workshop with radio, TV, and print journalists on reporting on bringing advanced maternal age and high parity into focus in Niger. Other workshop participants expressed interest reporting on the topic, noting advanced maternal age and high parity presented a new avenue for inquiry and mass sensitization. The I-Kit also helped MSI providers expand the topics they discuss with their clients. Because of the pilot, MSI reports they now integrate advanced maternal age and high parity into their provider and outreach trainings and daily outreach activities and are able to better tailor their reproductive health counseling to their at-risk clients. MSI mobile health agents and community-based health workers now inform their communities about advanced maternal age and high parity pregnancy risks and how to avoid or address them. The organization also shared the I-Kit with public-sector maternal and child health care providers to use and distribute.

In Togo, ATBEF providers found the I-Kit provider poster summarizing key counseling steps particularly salient and the infographic highlighting the urgency of addressing advanced maternal age and high parity to be useful in their community and group education sessions. ATBEF community health workers regularly rotate their discussion topics in community conversations; because of the I-Kit, they now include advanced maternal age and high parity pregnancy risks in that rotation. They found discussing the risks led women to share with others their personal experience with pregnancy or birth complications, realizing for the first time that age or parity may have played a role. Communities have been so receptive to the information and messages that ATBEF now includes advanced maternal age and high parity pregnancy risk information in all of its community health worker trainings. Clients participating in pilot activities even suggested that advanced maternal age and high parity information be shared in all communities and health facilities.

Communities in Togo have been so receptive to the Implementation Kit's information and messages that advanced maternal age and high parity pregnancy risk information is included in all community health worker trainings.

While the pilot was successful in producing adapted materials for use by each organization within their respective country, it did not allow for each material to be revised and reproduced, nor for data collection to measure actual behavior change among priority audiences. The pilot does, however, highlight lessons learned and recommendations on how MSI, ATBEF, and other similar organizations might adapt and prepare to use the I-Kit moving forward. Among them:

- **Create more image-centered materials for low-literacy clients.** While literacy is often higher in urban areas, rates are lower in smaller villages where advanced maternal age and high parity might be more prevalent. Replacing text with pictures of advanced maternal age and high parity complications would help non-literate clients to better retain necessary information.
- **Develop materials for delivering messages to large groups.** I-Kit materials, such as the counseling guides, infographics, and client brochures, were developed with one-to-one interactions in mind. Because much of MSI's and ATBEF's work involves community outreach and education sessions, they had a difficult time converting the counseling guides into a group discussion format. Creating a flip chart from these materials could be a worthwhile effort for organizations with similar portfolios.
- **Emphasize managing and planning to reduce advanced maternal age and high parity risks, rather than solely avoiding such types of pregnancies.** Participants in Niger especially questioned that 5 children was too many, and participants in both countries had difficulty accepting age 35 as an age to slow or stop childbearing. While the current I-Kit materials include information on the importance of seeking antenatal care and attended delivery for advanced maternal age and high parity pregnancy, they also encourage women to plan early to avoid having 5 or more children, or having children at age 35 or older. Any discussion of limiting childbearing is typically rejected in Niger. In such cases, adapted materials could more strongly emphasize recognizing and managing advanced maternal age and high parity pregnancy risks over outright pregnancy avoidance. Conversely, some women in urban Togo expressed desires to intentionally delay children to first pursue career and education goals. Here, tailoring materials to speak to younger women—perhaps university-aged—could help clients identify when they want to start or grow their families to avoid advanced maternal age pregnancy risks in the future.
- **Allow time for practice using new materials.** Both MSI and ATBEF implementers found the counseling guides replete with new or technical information, which was difficult to remember and assimilate at first. However, with practice, this information can become second nature. Both organizations appreciated the need to truly take the time to internalize the guides and practice with them to allow better-tailored counseling for clients.
- **Develop materials for men and revise terminology.** Especially in Niger, it was recommended that men and religious leaders be brought more into conversations about advanced maternal age and high parity pregnancy and family planning use. The I-Kit's brochures and counseling guides include men as secondary audiences and a guide for working with community-based groups highlights the importance of working with religious leaders. However, because these 2 groups are often family planning influencers or decision makers in pronatalist and conservative contexts such as Niger, developing modified counseling guides and brochures could more effectively engage them in conversation, involve them in changing harmful norms, and catalyze individual and community behavior change.

Messaging should emphasize managing and planning to reduce advanced maternal age and high parity risks, rather than solely avoiding such types of pregnancies.

## CONCLUSION

Our qualitative research and the Niger survey revealed that advanced maternal age and high parity pregnancies are linked to strong contextual and cultural factors in both Niger and Togo, and that family planning programs often do not sufficiently address the critical risks associated with pregnancies among women 35 years and older or those with 5 or more births. As shown in the Niger and Togo pilots, HC3's Healthy Timing and Spacing of Pregnancies I-Kit is a unique resource for governments, communities, service providers, women, and couples to learn and better communicate about advanced maternal age and high parity pregnancy dangers, and can provide the foundation for constructive change in beliefs, knowledge, and attitudes that perpetuate these risky pregnancies.

Since the I-Kit's launch, 2 additional projects (one in Cameroon, the other spanning multiple Latin American countries) have expressed interest in using the I-Kit in their context. Our pilot and adaptation activities, as well as these requests to use the materials, show that while discussing advanced maternal age and high parity pregnancy risks can be sensitive due to social and cultural taboos regarding birth limiting and modern contraceptive use, there is undeniable receptiveness to addressing these pregnancies in low- and middle-income countries. These opportunities should not be ignored and must be instead fanned as sparks to a momentous fire for action and constructive change.

## Supplementary Material

17-00417-Portillo-Supplement2.pdf

17-00417-Portillo-Supplement1.pdf

## Notes

**En français**

**L’âge maternel avancé à haut risque et la grossesse à parité élevée : s’attaquer à un besoin négligé par la recherche formative et l’action**

Des normes sociales néfastes et le manque de connaissances contribuent aux grossesses à risque chez les femmes âgées et à haute parité dans les pays à revenu faible et intermédiaire. Une ressource de communication pour le changement social et de comportement combinant des conseils techniques avec du matériel tangible pour les clients et les prestataires a été conçue pour traiter et prévenir de telles grossesses au Niger et au Togo.

**RÉSUMÉ**

La grossesse chez les femmes d’âge maternel avancé (35 ans ou plus) ou chez les femmes de grande parité (celles ayant eu 5 naissances ou plus) est liée à la mortalité maternelle et infantile. Pourtant, on sait peu de choses sur les causes de ces grossesses, car elles sont souvent négligées dans les programmes existants de planification familiale et de santé reproductive. Pour mieux comprendre le contexte dans lequel se produisent des grossesses chez les femmes d’âge avancé et de parité élevée et l’acceptabilité de discuter des risques associés, le Health Communication Capacity Collaborative (HC3) a mené une recherche qualitative formative entre janvier et mars 2015 sur la perception et les déterminants de ces grossesses dans les zones rurales et urbaines du Niger et du Togo. Nous avons complété cette recherche par des analyses secondaires de données provenant des Enquêtes Démographiques et de Santé et d’une enquête menée au Niger en 2014. Notre recherche formative a montré que les participants togolais urbains ont démontré plus de connaissances des risques de la grossesse à l’âge maternel avancé et à parité élevée que les participants au Niger dans son ensemble. Nous avons constaté que ces grossesses étaient généralement considérées comme faisant partie des normes de reproduction dans les situations où les taux de fécondité restent exceptionnellement élevés, en particulier au Niger. Les normes sociales, religieuses et de genre, y compris la compétition entre coépouses, ont également conduit les femmes à des situations de parité élevée et d’âge maternel avancé, notamment au Niger, soulignant la nécessité d’intégrer les hommes et les leaders communautaires et religieux dans les discussions de planification familiale afin d’accroître son acceptation. L’étude a également fourni des informations importantes nécessaires pour traiter ces grossesses à haut risque à travers des interventions de communication de santé culturellement appropriées. Une idée principale était que les prestataires avaient souvent des informations incomplètes sur les grossesses à l’âge avancé et les grossesses à parité élevée et manquaient de compétences en communication, de protocoles et d’outils pour discuter de ces risques de grossesse avec les clients. HC3 a utilisé ces résultats et d’autres pour créer un kit de mise en œuvre (I-Kit) pour les responsables de programmes de planification familiale et de santé maternelle et infantile, avec des conseils et des outils concrets pour faire face à la grossesse à l’âge maternel avancé et à parité élevée grâce à la communication sur le changement social et comportemental. Le I-Kit comprend du matériel de communication sur la santé pour engager les femmes, les hommes, les décideurs, les communautés, les prestataires de soins de santé, les journalistes et autres. En 2016 et 2017, une organisation au Niger et une au Togo ont piloté le I-Kit, intégrant des outils I-Kit sélectionnés dans leurs programmes uniques, et documentant leurs expériences. Les deux organisations ont crédité le I-Kit d’élargir la portée de leurs programmes pour aborder maintenant l’âge maternel avancé et la grossesse paritaire élevée et ont fourni des suggestions concrètes pour adapter le matériel en fonction de l’activité et du public visé.

## Notes

**Peer Reviewed**

**Funding:** This research would not have been possible without the generous support and funding from the United States Agency for International Development (USAID) under the Health Communication Capacity Collaborative Project.
