# Supplementary material for: High-Risk Advanced Maternal Age and High Parity Pregnancy: Tackling a Neglected Need Through Formative Research and Action
Source: Glob Health Sci Pract. 2018 Jun 27;6(2):372–83. doi: 10.9745/GHSP-D-17-00417 (PMC6024617; doi:10.9745/GHSP-D-17-00417)
Supplement: 17-00417-Portillo-Supplement2.pdf [file 17-00417-Portillo-Supplement2.pdf]

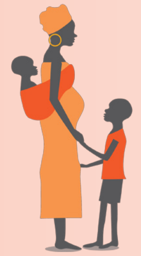

Healthy Timing and Spacing of Pregnancy

Case Study: SBCC Implementation Kit Helps Communities in Niger Communicate about High-Risk Pregnancies

April 2017

"The [Healthy Timing and Spacing of Pregnancy] pilot project brought to light new ways we can help women choose to protect themselves and their children from the known risks of AMA and HP pregnancy."

– Sylvie Ramandrosoa, MSI-Niger Country Director

HC3 and the HTSP I-Kit

The Health Communication Capacity Collaborative (HC3) is a five-year, global project funded by USAID to strengthen developing country capacity to implement state-of-the-art health communication programs. Among other health areas, HC3 works in family planning on topics such as healthy timing and spacing of pregnancy (HTSP).

Global HTSP activities have focused largely on preventing closely spaced and early (i.e., before age 18) pregnancies. Much less attention has been given to the dangers of having too many pregnancies, or having children later in life. In 2014, HC3 completed a desk review on knowledge and attitudes around advanced maternal age (AMA) and high parity (HP) pregnancy in low- and middle-income countries and an evidence review and secondary analyses of Demographic and Health Survey (DHS) AMA and HP Niger and Benin data. In 2015, HC3 conducted qualitative research and quantitative secondary analyses to understand the factors driving such risky pregnancies in Niger and Togo.

Based on this research, HC3 developed the HTSP AMA/HP Implementation Kit (I-Kit) to help program managers implement social and behavior change communication (SBCC) activities that address the neglected topic of AMA/HP pregnancy. The I-Kit, available in French and English, also includes a series of adaptable tools, including:

- a client brochure (one for less conservative audiences and one for more conservative audiences);
- a counseling and assessment guide for providers;
- a counseling and assessment guide for community health workers;
- a reminder poster for facility-based providers;
- a guide for journalists;
- a guide for researchers;
- AMA and HP infographics for policy and decision-makers; and
- a guide for working with community-based groups.

Introduction

Marie Stopes International (MSI) helps women to have children by choice, not chance. Invited into Niger by the Ministry of Health (MOH) in 2013, MSI-Niger offers high-quality reproductive health information and services in the regions of Niamey, Tillaberi and Maradi. MSI-Niger works primarily through a static clinic in Niamey, four mobile teams (i.e., a midwife, assistant and driver) in Tillaberi and Maradi, Marie Stopes Ladies (MS Ladies, i.e., mobile midwives sent to health posts and youth centers), social mobilization agents (Agents de Mobilization Sociale – AMS) and community-based promoters (Relais Communautaires – RECOs). Mobile teams provide services in MOH-operated health centers that would not otherwise provide birth spacing services. By expanding the method mix and increasing demand for and improving access to birth spacing services, MSI-Niger is helping the government achieve its goal of increasing the contraceptive prevalence rate (CPR) from 12.2 percent in 2012 to 50 percent by 2020.

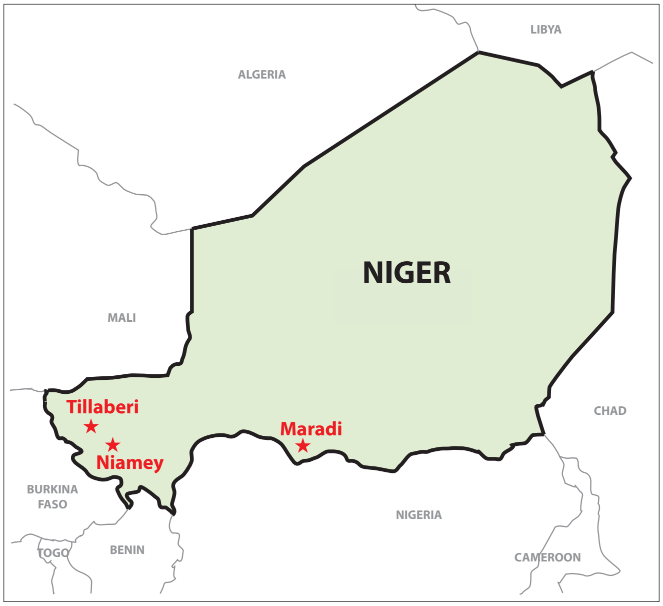

MSI-Niger provides quality reproductive health information and services in and around Niamey, Tillaberi and Maradi.

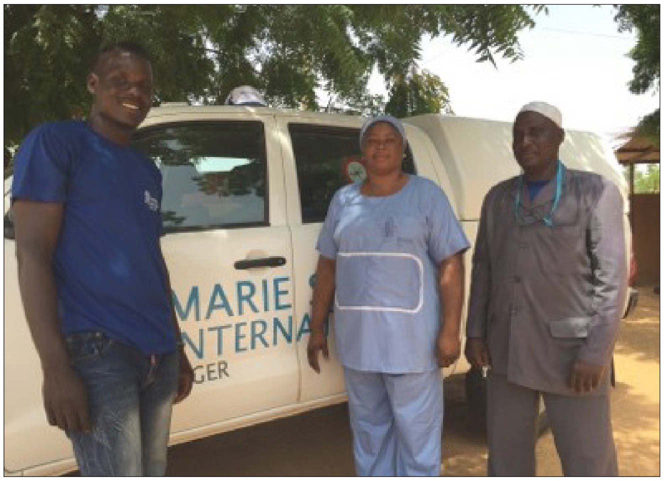

An MSI-Niger Mobile Team. © 2016, Carol Hooks. All rights reserved.

Fertility, AMA and HP in Niger

Niger has one of the highest maternal mortality rates in the world, at 553 maternal deaths per 100,000 childbirths (WHO et al., 2015), and the highest fertility rate, at 7.6 children per woman (Niger DHS, 2012; World Bank, 2017). Forty-three percent of women have given birth at least five times, and 60 percent have given birth at or after age 35 (HC3, 2014). AMA and HP pregnancies are common in both rural and urban areas and mostly perceived as normal, though AMA pregnancies in high-parity women and women with married daughters are somewhat less acceptable (HC3, 2016).

According to Niger's 2012 Demographic and Health Survey (DHS), over 50 percent of married women of reproductive age (WRA, ages 15 to 49) wish to delay their next pregnancy, despite the low CPR, which includes about four percent using the lactational amenorrhea method (LAM). Barriers to modern contraception and otherwise improved maternal health include religious beliefs tied to Islam and a culture that values large families. Niger is among the few countries where the desired family size is more than nine children. The notion of limiting births is widely rejected on religious grounds, and only nine percent of married WRA say they want no more children.

Using the HTSP I-Kit in Niger

In 2016, HC3 awarded MSI-Niger $5,000 to pilot the HTSP I-Kit over a four-month period (July to October 2016). MSI-Niger used this funding to help mobile providers, AMS, RECOs and journalists to communicate clear, simple AMA and HP messages as a way to increase adoption of modern contraceptive methods by at-risk women and couples. The core project team consisted of the Director of Operations and Programs in a supervisory role, the Social Mobilization Officer (SMO) responsible for day-to-day project management and an intern providing logistical support. The Communications and Marketing Manager supported training, and the Monitoring and Evaluation Coordinator supported data collection and reporting.

An HC3 consultant provided targeted virtual and on-site technical assistance (TA) consisting of Skype™ calls every two weeks, covering topics such as tool selection and printing, training plans, data collection tools, participant feedback, and overall progress. Because the team had social mobilization expertise but lacked SBCC experience, the consultant directed the SMO to relevant sections in the I-Kit and to training materials developed for an SBCC workshop in Haiti, *NPI: Documents pour la formation en CCSC*. The consultant spent one week in Niger toward the end of the project to observe, advise and document.

Based on what they thought would be most useful for their staff and audiences—and could test with the time and funding available—they chose to pilot the following tools:

HTSP I-Kit Tool	Used by	Used for
**1. Implementation manual for program managers**	Leadership team and SMO	Staff orientation
**2. Client brochure for more conservative audiences**	Mobile Teams, AMS, MOH Maternal and Child Health (MCH) providers	Client education, assessment and reminder
**3. Counseling and assessment guide for providers**	Mobile Team Family Planning (FP) Providers, MOH	Counseling, assessment and education
**4. Counseling and assessment guide for community health workers**	AMS	Community sensitization and individual pre-counseling
**5. Reminder poster for facility-based providers**	Mobile Team FP Providers, MOH	Client counseling and assessment
**6. Journalist guide**	• SMO<br>• Journalists	• Orienting radio, TV, print and online journalists<br>• Reporting

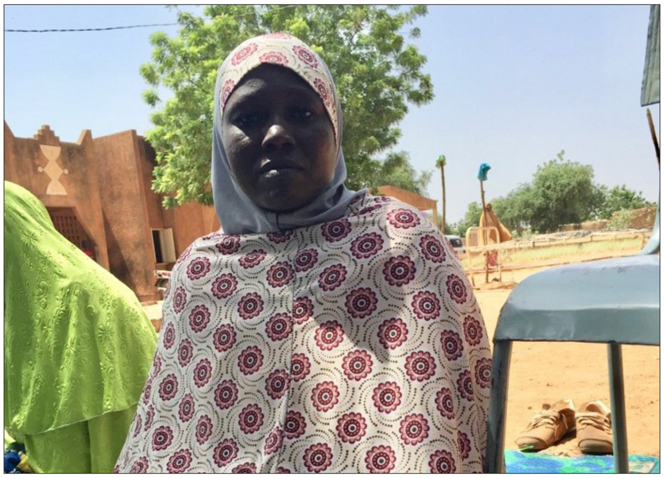

An AMA and HP woman in Niger. *© 2016, Carol Hooks. All rights reserved.*

MSI-Niger held a one-day workshop with eight journalists to gather their feedback on the journalist guide. This also served to orient journalists to AMA and HP pregnancy risks and to encourage them to report on it. Participants said the guide responded well to their needs and would be very helpful if they obtained funding to do the needed reporting. One TV and one print journalist reported on the August workshop, thereby calling attention to the topic. The pieces ran on Radio-Télévision Sarraounia and in the Enqueteur newspaper, respectively.

The project team used the I-Kit to orient managers and train five mobile providers and five AMS. AMS then trained RECOs. Mobile teams integrated AMA/HP counseling into their normal service provision. AMS discussed AMA and HP risks during their regular pre-counseling and group education sessions and with a limited number of community leaders. RECOs included discussion of AMA and HP during home visits. The 12 sites selected averaged 14 to 47 birth spacing clients older than 25, and nine to 34 birth spacing clients with at least four children. MSI-Niger staff hung the posters at the 12 intervention sites and other MSI-visited sites, and gave most of the brochures to the managers of health centers/posts to use and distribute as they saw fit. Activities took place in Niamey and the Kollo, Flingué and Say districts of Tillabery.

MSI-Niger held a one-day workshop with eight journalists to gather their feedback on the journalist guide. This also served to orient journalists to the topic and to encourage them to report on it. Participants said the guide responded well to their needs and would be very helpful if they obtained funding to do the needed reporting. One TV and one print journalist reported on the August workshop, thereby calling attention to the topic. The pieces ran on Radio-Télévision Sarraounia and in the Enqueteur newspaper, respectively.

The project team used the I-Kit to orient managers and train five mobile providers and five AMS. AMS then trained RECOs. Mobile teams integrated AMA/HP counseling into their normal service provision. AMS discussed AMA and HP risks during their regular pre-counseling and group education sessions and with a limited number of community leaders. RECOs included discussion of AMA and HP during home visits. The 12 sites selected averaged 14 to 47 birth spacing clients older than 25, and nine to 34 birth spacing clients with at least four children. MSI-Niger staff hung the posters at the 12 intervention sites and other MSI-visited sites, and gave most of the brochures to the managers of health centers/posts to use and distribute as they saw fit. Activities took place in Niamey and the Kollo, Flingué and Say districts of Tillabery.

> *"Family planning is a national preoccupation, but we don't know how to approach it. It is not in hiding things that one finds solutions."*
>
> – TV Journalist

Successes and What Worked Well

Of all the I-Kit components, the journalist guide and workshop provided project managers with the most immediate result and the most satisfaction. Radio, television and print journalists attended, participated actively and expressed interest in reporting on this important topic. They said AMA and HP presented a new avenue for inquiry and mass sensitization and that it would be key to engage religious leaders. As an outcome, they developed a list of types of interviewees and messages to develop for different audiences and media. At the end of the workshop, one television

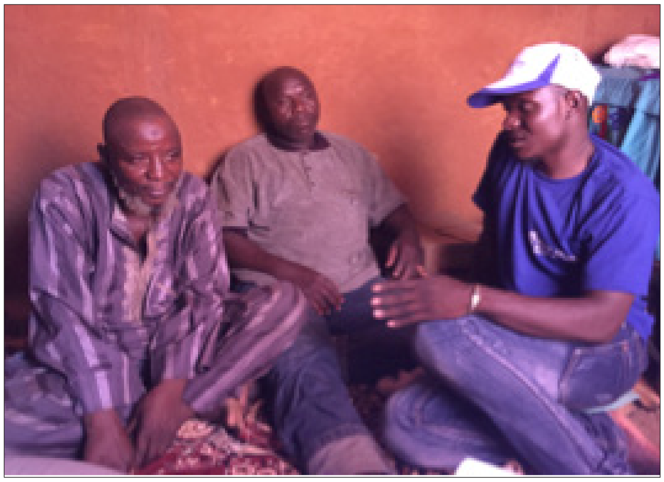

MSI-Niger AMS discusses AMA/HP with local imam. *© 2016, Carol Hooks. All rights reserved.*

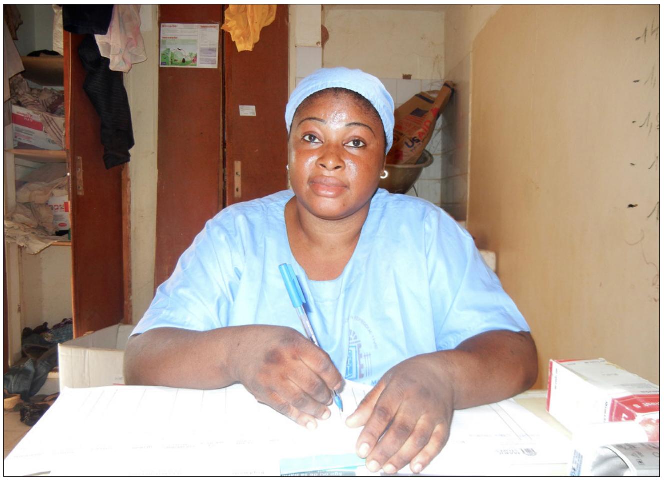

Healthcare provider in Niger, © 2016, Carol Hooks. All rights reserved.

journalist interviewed MSI-Niger's SMO and reported on the workshop and topic that same day.

Using the I-Kit helped MSI-Niger focus more on older and HP women and their needs. Prior to the I-Kit pilot, MSI-Niger providers rarely if ever spoke to clients about the risks of AMA and HP pregnancies. After using the I-Kit, MSI says they have integrated AMA and HP into day-to-day client outreach and they better tailor their counseling for at-risk women. AMS and RECOs also now inform communities about AMA and HP risks and ways to avoid or mitigate them. MSI-Niger shared I-Kit tools with MOH MCH providers to use and distribute and has incorporated AMA and HP information into its provider and outreach worker training and activities.

In all, MSI-Niger reported reaching more than 12,700 people with AMA/HP information or counseling during September and October 2016, with nearly 10,000 of those reached through large group education sessions, often held on market day in implementation communities. The table below shows the number of people in each audience segment reached by each cadre during the two month period.

Lessons Learned

Due to time and funding constraints, MSI-Niger was unable to redesign or adapt the materials before integrating them into their activities. In using the I-Kit and tools as-is, however, MSI-Niger identified several ways to adapt the I-Kit for the Niger context:

- **Adapt materials for low-literacy clients.** Seventy percent of Niger's population is not literate, and the proportion in rural areas is even higher. AMS and mobile team providers reported anecdotally that audiences found the content interesting but were not able to perform the self-assessment included in the client brochure as it is very text-based. Including more pictures – especially of AMA and HP complications – in the client brochure would make it easier to educate and motivate non-literate audiences.
- **Develop materials for delivering messages to large groups.** The MSI-Niger AMS hold many more large group discussions than one-to-one counseling sessions. The counseling guides and client brochures, for example, were designed with a one-to-one, provider-client or -couple approach in mind, but MSI saw they could adapt this content to flip chart pages showing images of the complications, and a guide for holding discussions on AMA/HP with large groups of

	**Number of People Reached in Each Audience Segment (Sept 2016 – Oct 2016)**					
	**Cadre**	Women < 25[1]	Women > 25	Men < 25	Men > 25	Total
September 2016	AMS	1,686	2,202	643	545	5,076
	Provider, Mobile Team 1	137	196	0	0	333
	Provider, Mobile Team 2	79	166	0	0	245
	Community Relays	134	327	59	87	607
	**Total Reached**	2,036	2,891	702	632	**6,261**
October 2016	AMS	1,889	1,606	559	236	4,290
	Provider, Mobile Team 1	150	278	0	2	430
	Provider, Mobile Team 2	146	301	0	0	447
	Community Relays	521	623	70	115	1,329
	**Total Reached**	2,706	2,808	629	353	**6,496**
	**TOTAL**	**4,742**	**5,699**	**1,331**	**985**	**12,757**

[1] Because MSI-Niger's portfolio includes a youth focus, individuals reached are routinely categorized as being older or younger than age 25, their age limit for who is considered "youth."

women. The pages could also be added to existing resources, such as MSI's FP flip chart.

- **Revise the terminology.** To better align with Niger's cultural and religious norms, "birth spacing" should be used instead of "family planning."
- **Develop materials for men and religious leaders.** Given Niger's strong adherence to cultural and religious traditions, men and religious leaders need a special focus in birth spacing discussions. For example, additional modules on reaching men and religious leaders could be added to the journalist guide, and MSI could create a relevant discussion guide for AMS and RECOs. Also, the client brochure could be adapted (or a new brochure created) to reach men and religious leaders.
- **Plan for more training and practice sessions to cover new material.** Since topics such as antenatal care and nutrition were new to AMS in particular, training sessions on these topics and time to build skills in practice sessions using the materials (e.g., guides and client brochure) would be helpful.
- **Help providers develop skills to tailor messages to individual clients.** MSI providers and AMS would benefit from skills-building sessions to learn how to tailor the information to individual clients instead of trying to follow the "script" provided in the counseling guide. Providers found it impractical to follow the counseling guide verbatim with clients, but with practice were able to pull key points into their client sessions. Streamlining the provider counseling guide, providing examples of how to integrate AMA/HP into a typical counseling session and adding a one-page summary or counseling algorithm could make the content easier to integrate into what MSI's providers already do.

Conclusion

MSI-Niger used the I-Kit to stimulate discussion and reflection about two known but under-appreciated contributors to maternal and infant morbidity and mortality – AMA and HP pregnancies. In doing so, MSI-Niger expanded its outreach to women over age 25 and found an appropriate entrée for engaging men and religious leaders. The content and structure of the I-Kit helped MSI-Niger enhance communication with communities – including through mass media – and with women and couples at risk. Adapting I-Kit tools and SBCC approaches based on this experience could further improve MSI-Niger's ability to help to satisfy Niger's unmet need for birth spacing.

References

Bongaarts, J. (2016). WHO, UNICEF, UNFPA, World Bank Group, and United Nations Population Division Trends in Maternal Mortality: 1990 to 2015 Geneva: World Health Organization, 2015. *Population and Development Review*, 42(4), 726-726.

Health Communication Capacity Collaborative (HC3). (2014). *Engaging Families for Healthy Pregnancies – A Focused Desk Review of Knowledge, Attitudes and Behaviors Related to Pregnancies in Three High-Risk Situations: Advanced Maternal Age; High Parity; and Rapid Repeat Pregnancies After Abortion or Miscarriage.* Baltimore: Johns Hopkins Center for Communication Programs.

Health Communication Capacity Collaborative (HC3). (2016). *Qualitative Research on Advanced Maternal Age and High Parity Pregnancies in West Africa*. Baltimore: Johns Hopkins Center for Communication Programs.

Hooks, Carol. (2017). *HTSP I-Kit Localization Summary Report*. Unpublished manuscript.

Institut National de la Statistique (INS) & ICF International. (2013). *Enquête Démographique et de Santé et à Indicateurs Multiples du Niger 2012*. Calverton, Maryland, USA: INS and ICF International.

Marie Stopes International (MSI). (2017). *What We Do*. Retrieved from https://mariestopes.org/what-we-do/

Marie Stopes International-Niger. (2016). *Rapport Final du Projet Pilote du Kit PEIGS*. Unpublished manuscript.

The World Bank. (2017). *Fertility rate, total (births per woman)*. Retrieve from http://data.worldbank.org/indicator/SP.DYN.TFRT.IN?year_high_desc=true
