# Supplementary material for: High-Risk Advanced Maternal Age and High Parity Pregnancy: Tackling a Neglected Need Through Formative Research and Action
Source: Glob Health Sci Pract. 2018 Jun 27;6(2):372–83. doi: 10.9745/GHSP-D-17-00417 (PMC6024617; doi:10.9745/GHSP-D-17-00417)
Supplement: 17-00417-Portillo-Supplement1.pdf [file 17-00417-Portillo-Supplement1.pdf]

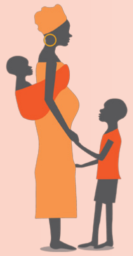

Healthy Timing and Spacing of Pregnancy

Case Study: SBCC Implementation Kit Helps Providers Address High-Risk Pregnancy in Togo

April 2017

"We used to say, 'the number you want, when you want.' Now we realize we have to nuance that message and make couples aware of risks they might face if they have too many [pregnancies] or too late."

– Anita Kouvahey-Eklu, ATBEF HTSP Project Coordinator

HC3 and the HTSP I-Kit

The Health Communication Capacity Collaborative (HC3) is a five-year, global project funded by USAID to strengthen developing country capacity to implement state-of-the-art health communication programs. Among other health areas, HC3 works in family planning on topics such as healthy timing and spacing of pregnancy (HTSP).

Global HTSP activities have focused largely on preventing closely spaced and early (i.e., before age 18) pregnancies. Much less attention has been given to the dangers of having too many pregnancies, or having children later in life. In 2014, HC3 completed a desk review on knowledge and attitudes around advanced maternal age (AMA) and high parity (HP) pregnancy in low- and middle-income countries and an evidence review and secondary analyses of Demographic and Health Survey (DHS) AMA and HP Niger and Benin data. In 2015, HC3 conducted qualitative research and quantitative secondary analyses to understand the factors driving such risky pregnancies in Niger and Togo.

Based on this research, HC3 developed the HTSP AMA/HP Implementation Kit (I-Kit) to help program managers implement social and behavior change communication (SBCC) activities that address the neglected topic of AMA/HP pregnancy. The I-Kit, available in French and English, also includes a series of adaptable tools, including:

- a client brochure (one for less conservative audiences and one for more conservative audiences);
- a counseling and assessment guide for providers;
- a counseling and assessment guide for community health workers;
- a reminder poster for facility-based providers;
- a guide for journalists;
- a guide for researchers;
- AMA and HP infographics for policy and decision-makers; and
- a guide for working with community-based groups.

Introduction

The Association Togolaise pour le Bien-Être Familial (ATBEF), an International Planned Parenthood Federation member, has been improving sexual and reproductive health in Togo since 1975. Operating through five clinics, two mobile teams and community health workers (CHWs) (a subset of whom provide injectable contraceptives), ATBEF provides comprehensive women's health services. ATBEF delivers family planning, maternal health and gynecological services, as well as HIV prevention, testing and management for all ages and genders.

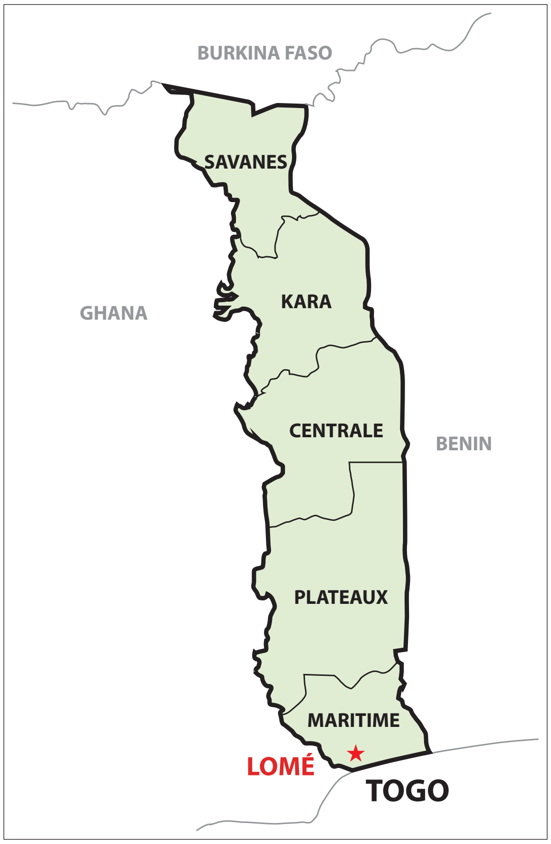

ATBEF provides high quality sexual and reproductive health information and services throughout Togo, with clinics in the Maritime, Plateaux, Centrale and Kara regions.

Fertility, AMA and HP in Togo

According to its most recent Demographic and Health Survey (DHS) (Togo DHS 2013 - 2014), Togo's maternal mortality rate is 401 maternal deaths per 100,000 childbirths. Women in Togo have an average of 4.8 children, with fertility lower in Lomé (3.5) and highest in the north (6.0). Forty-six percent of women have given birth at or after the age of 35, and 22 percent have given birth to five or more children.

Thirty-two percent of married women of reproductive age (WRA, ages 15 to 49) want no more children, and 37 percent want to delay their next pregnancy by at least two years. Seventeen percent use modern contraception. Respondents during qualitative research conducted in Togo in 2015 cited low knowledge levels, illiteracy, poverty and religious beliefs as reasons for resistance to modern contraceptive method use.

Using the HTSP I-Kit in Togo

HC3 awarded ATBEF $5,000 to pilot the HTSP I-Kit. From September 2016 to March 2017, ATBEF assessed the use of selected tools from the I-Kit in its main clinic, located in Togo's capital, Lomé, and in four communities in Avé District (about 40 kilometers northwest of Lomé). ATBEF used the I-Kit to strengthen the SBCC capacity of providers and CHWs to prevent and address AMA and HP pregnancy and to organize monthly group education sessions for clinic- and community-based clients and audiences.

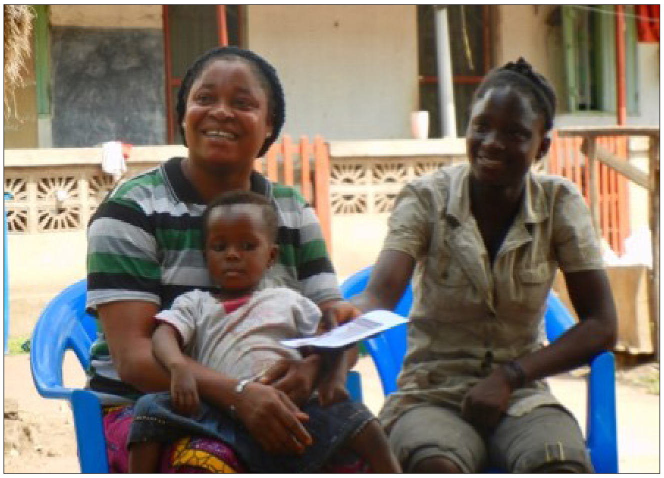

AMA/HP community discussion participants, Avé District, Togo. © 2016, Carol Hooks. All rights reserved.

ATBEF's Community Outreach/Learning Center/HIV Program Manager led the organization's I-Kit pilot team, supported by its Medical Officer and Communications Officer. The Monitoring, Evaluation and Research Officer and the Program Director validated project monitoring tools. While ATBEF had extensive experience with information, education and communication (IEC) as well as behavior change communication, SBCC—which incorporates social norms and environmental factors into the health communication design and implementation process—was new to them.

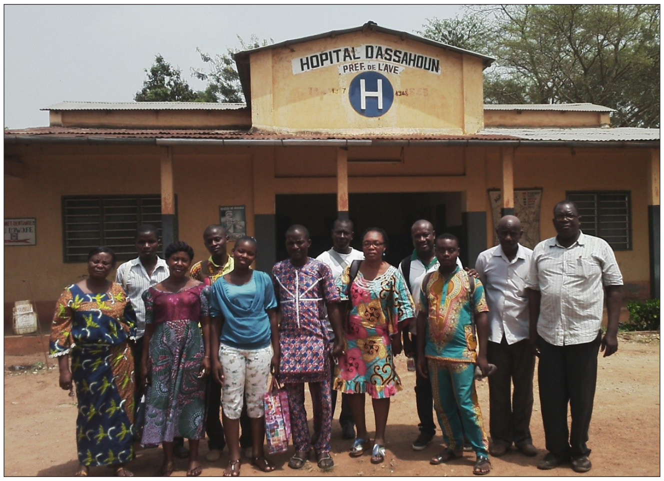

ATBEF's Avé District CHWs, AMA/HP pilot project management team, and Maritime Regional Coordinator, following the project wrap-up meeting, March 2017. © 2017, ATBEF.

To support ATBEF's need for more grounding in SBCC, HC3 supplied the following links to French-language SBCC resources alongside the I-Kit:

- Acces des Communautes aux Contraceptifs Injectables: Un Guide Pour le Plaidoyer
- Un Film de Plaidoyer: «l'Accès à base communautaire à la contraception injectable: du simple bon sens»
- Les Ressources Pour l'Action
- NPI: Documents pour la formation en CCSC
- Jhpiego: cours en français

An HC3 consultant provided additional SBCC resources and targeted virtual and on-site technical assistance (TA) consisting of telephone calls every two to three weeks. These calls covered topics such as tool selection and printing, training plans, data collection tools, participant feedback and overall progress. The consultant spent one week in Togo halfway through the project to observe, assist and document.

Based on what ATBEF thought would be most useful for their staff and audiences—and could test with the time and funding available—they chose to pilot the following tools:

HTSP I-Kit Tool	Used by	Used for
**1. Implementation manual for program managers**	Project Coordination	Managers and Ministry of Health
**2. Client brochure for less conservative audiences**	Providers and CHWs	Client reminder (post-visit)
**3. Counseling and assessment guide for providers**	Facility-based providers	Client counseling, assessment and education
**4. Counseling and assessment guide for community health workers**	CHWs	Community education / sensitization
**5. Reminder poster for facility-based providers**	Facility-based providers	Client counseling and assessment
**6. Infographics for policy and decision-makers**	Project Coordinator and CHWs	Community education sessions

ATBEF used the I-Kit to train 15 providers and 10 CHWs, and to orient the management team and a Ministry of Health (MOH)/Maternal and Child Health Department representative on AMA and HP pregnancy. Including the MOH in the HTSP orientation facilitated community-level work and resulted in buy-in for expanding the message to MOH service providers.

Provider and CHW training lasted three days each and included presentation, discussion, role-play and practice with the I-Kit brochure and counseling guides. Provider training included a full day of practice in ATBEF's central clinic.

CHW training took place at the Health Directorate in Avé. Participants included ATBEF's regional coordinator, 10 CHWs from four Avé villages (Djégbakondji, Atti-touwi, Yoto and Tiviepé) and a CHW coordinator. The District Health Director opened the workshop, stressing the importance of both the topic and the role of CHWs. As practice, CHWs led one group session with community members and one with community leaders.

Providers of family planning (FP), antenatal care (ANC), post-natal care (PNC) and HIV services integrated AMA and HP assessment and counseling into their day-to-day client interactions. They held 19 AMA- and HP-focused group education sessions with waiting clients, and counseled 542 clients on the risks of AMA and HP pregnancy. CHWs discussed AMA and HP during 139 home visits and 39 community discussions. These sessions defined AMA and HP, highlighted the increased risk of complications, promoted the use of modern FP methods to prevent risky pregnancies and addressed concerns about FP method side effects.

ATBEF also held three focus group discussions (FGDs): one each with male and female members of a family well-being group called Mognongnon (happy family), and one with five couples. Mognongnon group members are parents and community leaders and take

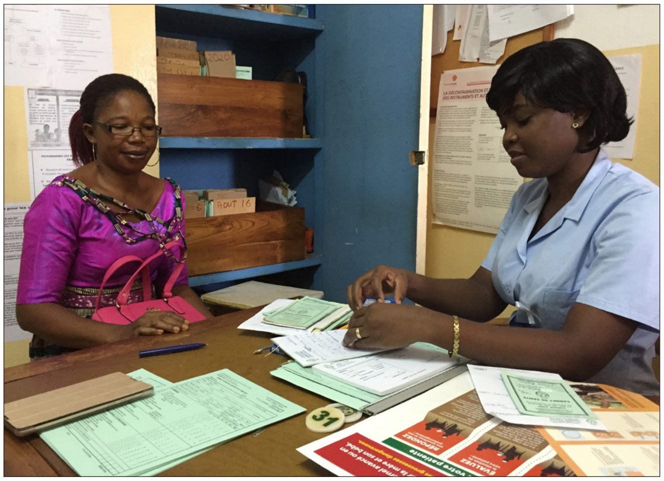

Counseling session, ATBEF Clinic, Lomé. © 2016, Carol Hooks. All rights reserved.

ATBEF Provider Training Topics

- HTSP
- Definition of AMA and HP pregnancy
- Importance of integrating AMA and HP concepts into daily clinic activities
- Risks associated with AMA and HP pregnancy
- SBCC
- Presentation of the HTSP tools and data collection forms
- Practice in the clinic conducting AMA and HP education sessions and integrating AMA and HP into FP, antenatal/post-natal care and HIV counseling sessions

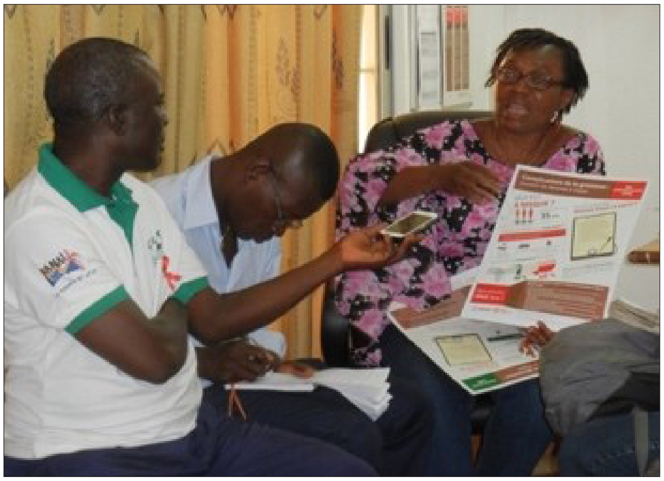

Focus group with male Mognongnon members, Lomé, Togo. *© 2016, Carol Hooks. All rights reserved.*

what they learn from Mognongnon to their homes, workplaces and communities. They include, among others, journalists, peer educators, business owners, and religious leaders. The couples group observed that in most situations the man decides how many children they will have – without considering the woman's age or parity. The participants in all three groups had been unaware of increased risks due to age and parity, and some reported having experienced the complications highlighted. Participants appreciated the information and materials and vowed to inform their communities.

During the question and answer portion of many group sessions, participants focused on women who delay childbirth to pursue education or careers, or because their husbands work elsewhere, and questioned the idea of 35 being too old for giving birth.

Successes and What Worked Well

ATBEF staff and partners appreciated highlighting AMA and HP women as key audiences and the new perspective about the role of community norms in behavior change. ATBEF liked the HTSP I-Kit content and structure and found it comprehensive and useful for learning about HTSP, AMA/ HP and SBCC. Staff expressed appreciation of SBCC concepts such as engaging influencing audiences, since they had not included them in their work before. ATBEF providers found the poster to be a useful reminder and the infographics an easy-to-access outline, list of risks and interesting statistics that made it very useful even for community group discussions. ATBEF staff used the I-Kit effectively to improve outreach and counseling with women and couples at risk.

The ATBEF training built on what participants did in their day-to-day work, rather than adding to it, and included practice sessions with feedback. For example, ATBEF CHWs regularly rotate community group discussion topics. Because of the I-Kit and their training, CHWs were able to include AMA and HP pregnancy into their rotation, and dedicate 30 to 60 minutes on just that topic (rather than featuring it as one topic among many). This made it more likely that community members understood the information. Clients and communities received the messages well, so ATBEF has incorporated AMA and HP pregnancy avoidance in all of its provider and CHW training.

The group and community discussion also elicited testimonials of having experienced complications and not realizing that age or parity might have contributed to them. The sessions inspired questions on the ideal age for men and women to have children, contraceptive methods, how to engage men in this conversation and how to help AMA women who want to conceive. Pilot participants found the topics so valuable that they suggested sharing AMA and HP messages in all communities and health facilities. In all, ATBEF reached more than 3,000 individuals with their I-Kit activities (detailed in the table at the top of page 5).

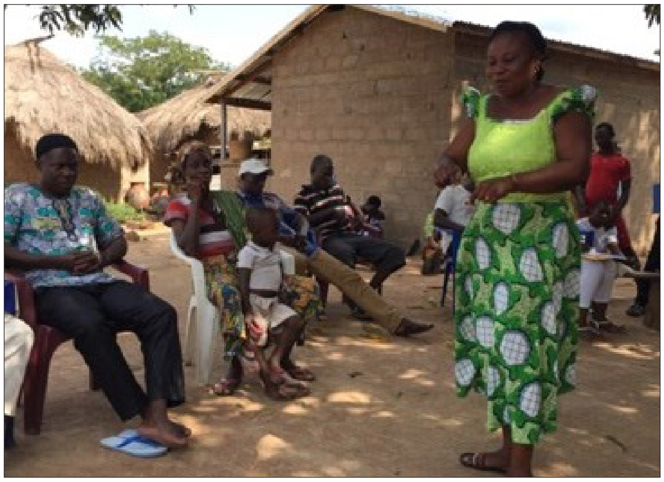

*ATBEF CHW answers community members' questions, Avé District . * *© 2016, Carol Hooks. All rights reserved.*

Lessons Learned

At the end of the project, ATBEF held a feedback session with partners, providers and CHWs. Many found the project to be a wake-up call. They said they learned that they needed to better tailor their approaches based on, for example, age, parity, community norms and behavior change theory. Among their key observations was that it will be difficult to limit births after age 35, but that they will insist on rigorous follow-up in those cases to improve health outcomes for mother and child.

Based on the pilot experience, ATBEF identified several "lessons learned" and ways to adapt the I-Kit:

Persons Reached through ATBEF CHW Community Discussions and Home Visits (Nov 2016 – Mar 2017)

Audience	Women (age 24+)					Men		Youth		Total
	AMA	HP	AMA + HP	Not AMA or HP	Unknown	Married	Single	Female	Male	
Number of participants	158	117	167	723	75	307	145	190	108	1,990

Persons Reached by ATBEF Clinic-Based Providers (Nov 2016 – Mar 2017)

Audience	Women (age 24+)					Men		Youth		Total
	AMA	HP	AMA + HP	Not AMA or HP	Unknown	Married	Single	Female	Male	
Number of participants	339	203	185	305	52	144	45	51	23	1,347
**TOTAL**										**3,337**

- **Adapt the materials for low-literacy clients.** While 66.5 percent of adults in Togo are literate, literacy is higher in cities and lower in some villages. Replacing text with pictures of AMA- and HP-associated complications would help low- and non-literate clients better understand and remember necessary information about these high-risk pregnancies.
- **Develop materials for delivering messages to large groups.** Because much of ATBEF CHWs' work involves community discussions, the CHWs repurposed the infographic – designed for use with health decision-makers at the national, district or clinic level – as a group education tool. Replacing the calls to action in the "What Can You Do" section of the tool with a list of FP methods could help remind CHWs what to cover in their talks.
- **Further simplify the counseling guides.** While both the provider and CHW guides include AMA and HP health risk definitions, sometimes this was too much information to serve as a quick-reference. Once health workers are familiar with the definitions themselves, adding a new table showing AMA risks, HP risks, and risks common to both could give the health workers the reminders they need at a glance.
- **Emphasize managing AMA and HP risks.** Although the I-Kit materials currently include information on the importance of seeking ANC and attended delivery during AMA and HP pregnancies, the I-Kit materials also encourage women and couples to plan their families to avoid these pregnancies. Participants in Togo were particularly troubled by the suggestion that age 35 was too old to have children, both because of social norms and the increasing number of women who wish to delay having children until after they have completed school or career goals. In such cases, adapted materials could more strongly emphasize the need for close follow-up and facility delivery for at-risk pregnancies.
- **Rethink "interactive" components.** As designed, the client brochure features an AMA and HP risk self-assessment, asking the woman questions such as, "Do you want to have a baby in the next two years? Are you 35 or older? Have you had at least five births?" Women in Togo had difficulty understanding this approach, making it less useful. This section could be replaced with a table listing AMA risks, HP risks and risks common to both.

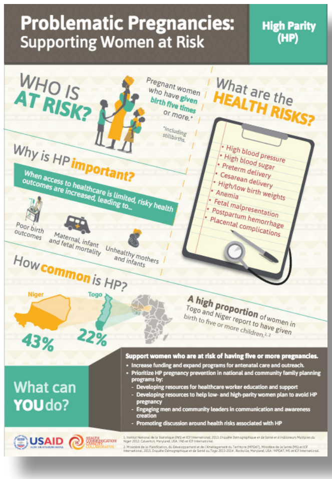

I-Kit HP infographic with "What Can You Do" section at bottom

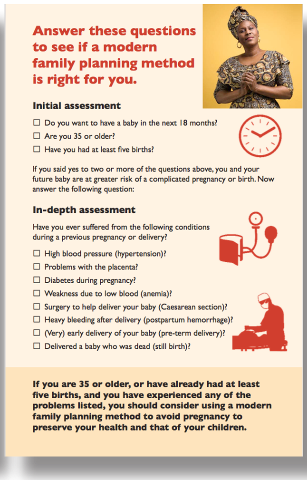

Client brochure page with the self-assessment

- **Allow time for practice using new materials.** ATBEF CHWs and providers initially found the counseling guides long, technical and time-consuming or difficult to integrate into their normal routines. However, once they fully assimilated the information and understood they were not expected to use the guide verbatim, both groups were able to adapt and individualize their approach for clients and group discussions. Scheduling practice sessions with volunteer couples, and practicing what to say to AMA and HP women already using contraception (e.g., stressing the importance of method continuation and recommending long-lasting methods), would help health workers feel more prepared for real-life scenarios.

Conclusion

Participation in the HTSP I-Kit Pilot project expanded how ATBEF thinks about its audiences and key messages. While managers and providers were generally aware that AMA and HP pregnancies were higher risk, they did not use these messages to motivate women and couples to adopt modern contraception to protect against these risks. ATBEF also had not been exposed to key SBCC concepts, such as the role of influencing audiences on women and their ability to adopt FP. The pilot provided a timely opportunity for ATBEF to reinvigorate its communication approach. Since the pilot, ATBEF has integrated HTSP and SBCC into all of its provider and CHW training—including training of MOH providers, thereby expanding the reach and influence of this small project. Additionally, they will incorporate the topic in future projects, such as upcoming work with university students where they will now encourage them to plan to avoid AMA pregnancy.

References

Health Communication Capacity Collaborative (HC3). (2014). *Engaging Families for Healthy Pregnancies – A Focused Desk Review of Knowledge, Attitudes and Behaviors Related to Pregnancies in Three High-Risk Situations: Advanced Maternal Age; High Parity; and Rapid Repeat Pregnancies After Abortion or Miscarriage.* Baltimore: Johns Hopkins Center for Communication Programs.

Health Communication Capacity Collaborative (HC3). (2016). *Qualitative Research on Advanced Maternal Age and High Parity Pregnancies in West Africa*. Baltimore: Johns Hopkins Center for Communication Programs.

Hooks, Carol. (2017). *HTSP I-Kit Localization Summary Report*. Unpublished manuscript.

Kouvahey-Eklu, Anita. (2017). Pr*ojet : Prévention des Grossesses Chez les Femmes en Age Avancé et à Haute Parité*. Unpublished manuscript.

Ministère de la Planification, du Développement et de l'Aménagement du Territoire (MPDAT), Ministère de la Santé (MS) & ICF International. (2015). *Enquête Démographique et de Santé au Togo 2013-2014*. Rockville, Maryland: MPDAT, MS and ICF International.
